# Incomplete peripheral retinal vascularisation in retinopathy of prematurity: is it the consequence of changing oxygen saturation?

**DOI:** 10.3389/fped.2023.1203068

**Published:** 2023-06-21

**Authors:** Sigal Zmujack Yehiam, Samantha K. Simkin, Rasha Al-Taie, Maisie Wong, Malcolm Battin, Shuan Dai

**Affiliations:** ^1^ Department of Ophthalmology, New Zealand National Eye Centre, Faculty of Medical and Health Sciences, The University of Auckland, Auckland, New Zealand; ^2^ Neonatal Intensive Care, Counties Manukau District Health Board, Auckland, New Zealand; ^3^ Neonatal Intensive Care, Newborn Services, Auckland City Hospital, Auckland, New Zealand; ^4^ Children’s Health Queensland Hospital and Health Service, Queensland Children’s Hospital, Brisbane, QLD, Australia; ^5^ Faculty of Medicine, The University of Queensland, Brisbane, QLD, Australia

**Keywords:** avascular retina, retinopathy of prematurity, oxygen saturation, normal retina vascularisation, treatment avascular retina in ROP, risk factors

## Abstract

**Background:**

We wish to determine the prevalence and risk factors of incomplete peripheral avascular retina (IPAR) in children screened for retinopathy of prematurity (ROP) and its association with oxygen saturation (SpO_2_) targets.

**Methods:**

A retrospective review of retinal images of premature infants born and screened for ROP in Auckland Region, New Zealand, between January 2013 and December 2017 was conducted. Images were reviewed to determine if avascular retina was present at their final ROP screening. The prevalence of peripheral avascular retina was compared among infants born prior to (Group 1) and after (Group 2) 2015 when the SpO_2_ target was increased. Infants with any concurrent ocular pathology or who had received ROP treatment were excluded.

**Results:**

In total, 62 (12.8%) of the total of 486 infants (247 in Group 1; 239 in Group 2) were found to have IPAR at their last ROP screening. Group 1 had more statistically significant infants with IPAR compared to Group 2 (39/247 infants and 23/239 infants respectively; *p* = 0.043).

**Conclusions:**

Incomplete peripheral retinal vascularisation occurred at a prevalence of 12.8% in infants at risk of ROP. Higher SpO_2_ targets did not increase the prevalence of incomplete peripheral retinal vascularisation. Low gestational age and low birth weight are likely risk factors for the development of avascular retina. Further research into the risk factors associated with incomplete peripheral retinal vascularisation and the associated long-term outcomes is needed.

## Introduction

ROP is a retinal vascular proliferative disease affecting premature infants with low birth weight and gestational age. Globally, it is estimated that at least 50,000 children each year are blind or severely visually impaired from ROP and it remains the leading cause of treatable childhood blindness (1). Among the multiple factors potentially contributing to the development of ROP, low gestational age, lower birth weight, and uncontrolled poorly regulated oxygen use are considered the main risk factors (2–4).

The relationship between ROP and hyper oxygenation is well recognised with the first epidemic of ROP being described in 1942 (5). At that time, the care of newborn infants included the use of unrestricted oxygen, which resulted in oxygen toxicity of the developing retina. Oxygen monitoring was subsequently introduced and refined to ensure that oxygen saturation remained within a defined range (6–8). Subsequently, several research studies were undertaken to refine these limits, collectively reported as the Neonatal Oxygenation Prospective Meta-analysis (9) (NEOPROM). This work reported that a lower SpO2 target (85%–89%) compared to a higher SpO2 target (91%–95%) was associated with an increased relative risk of mortality and necrotising enterocolitis but a decreased relative risk of severe retinopathy of prematurity (9). Therefore, there was a shift in neonatal practice with SpO2 targeted at 90%–95% for infants less than 28 weeks gestational age. These higher SpO2 targets were introduced in neonatal intensive care units (NICUs) throughout New Zealand in 2015.

Incomplete peripheral retinal vascularisation was first reported in ICROP 1987 by Aaberg et al. (10), and in recent years we have encountered increased numbers of premature infants with incomplete peripheral retinal vascularisation at term (11). The primary aims of this study were to determine the prevalence of incomplete peripheral retinal vascularisation in children screened for ROP in Auckland, New Zealand, over a 5-year period. The secondary aim was to identify potential risk factors for this observation by analysing demographic and neonatal health data. In particular, the association between a higher SpO_2_ target and the presence of incomplete peripheral retinal vascularisation was assessed.

## Methods

Infants included in this study had been screened for ROP across the four NICUs in Auckland, New Zealand, between January 2013 and December 2017. In New Zealand, all premature infants who are born ≤30 weeks gestational age or weighing ≤1250 g, or selected premature infants ≥1,250 g and ≥30 weeks with unstable clinical course who are deemed at high risk according to their attending neonatologist, are routinely screened for ROP (12).

All premature infants were screened using the RetCam digital imaging system (Clarity Medical Systems Inc., Pleasanton, CA, USA) with a 130-degree lens to capture images of the posterior pole and nasal and temporal peripheral retina through a dilated pupil. In our hospitals, Retcam digital imaging is the standard method for ROP screening. A Retcam retinal fluorescein angiogram is performed in ROP cases where there is clinical concern of significant area of avascular retina based on coloured Retcam retinal images. Retinal images captured at last screening (between 37 and 45 post menstrual weeks) for all infants that met ROP screening criteria prior to discharge from active ROP screening were reviewed and analysed by two paediatric ophthalmologists experienced in ROP screening (12). Incomplete peripheral retinal vascularisation is defined as any area of the retina that lacks retinal vasculature with no active ROP disease. This area was further defined by location and clock hours. ROP if present was classified according to the international classification of ROP (ICROP) (13–15).

Due to the change in SpO_2­_ target, in 2015, two groups of children were defined. Group 1 was children born and screened from 1 January 2013 to 31 December 2014 who were exposed to lower SpO_2_ (88%–92%). Group 2 had a higher SpO­_2_ targets (90%–94%) included all children born and screened from 1 January 2016 to 31 December 2017. Children born and screened in 2015 were excluded to allow for a wash out period. Premature infants with concomitant ocular disease or who had undergone any treatment for ROP were excluded.

This study was approved by the National Health and Disability Ethics Committee (14/NTA/183) and abided by the declaration of Helsinki.

### Statistical analysis

Statistical analysis was completed with IBM SPSS software version 22 (Armonk, New York, United States of America). Descriptive statistics for infant demographics and characteristics were calculated as mean ± standard deviation. For nonparametric data, the median and interquartile range were determined. Differences between groups were calculated by Pearson Chi-squared test for categorical data, independent sample t-test for continuous data, and the Mann–Whitney *U* test for non-parametric variables.

## Results

A total of 486 premature infants were screened for ROP in four NICUs during the study period. Median (range) gestational age was 28.0 (23–34) weeks and median (range) birth weight was 1,110 (450–2,085) grams. The ethnicity distribution of infants was 37% European, 22% Asian, 20.6% Pacific peoples, 17.7% New Zealand Māori, and 2.7% other ethnicities. The baseline demographics were similar in both groups: males comprised 54.5% (no = 265) of the cohort and further demographic data is displayed in Table 1. ROP characteristics of all the patients are shown in Table 2. The total number of premature infants who developed ROP was similar between the two groups (54.6% vs. 53.5% *p* = 0.808). No ROP was detected in 45.9% of the premature infants, stage 1 ROP was diagnosed in 27.2%, stage 2 ROP in 23.2%, and stage 3 ROP in 3.7% of the premature infants. The location of ROP, at its worse stage in the disease process, was predominantly (84.4%) in zone 2 followed by 15.2% in zone 3 and 0.4% in zone 1.

**Table 1:** Demographic data—all patients.

	Group 1 Low SpO_2_ target	Group 2 High SpO_2_ target	Total	*p*-value
Total *n* (%)	247 (50.8%)	239 (49.2%)	486 (100%)	
Male *n* (%)	131 (53.0%)	134 (56.1%)	265 (54.5%)	0.502
Female *n* (%)	116 (47%)	105 (43.9%)	221 (45.5%)	
GA in weeks median (IQR)	28 (26–30)	28 (27–29)	28 (27–29)	0.915
BW in grams median (IQR)	1,110 (880–1,320)	1,110 (930–1,275)	1,110 (910–1,290)	0.830
PMA at worst ROP median (IQR)	33 (32–35)	33 (32–35)	33.0 (32–35)	0.639

GA, gestational age; BW, birth weight; PMA, post-menopausal age; IQR, interquartile range.

**Table 2:** The ROP features of all patients.

ROP stage		Group 1 Low SP0_2_		Group 2 High SPO_2_		Total	*p-*value
		*n*	%	*n*	%	*n* (%)	
	Stage 0	112	45.3	111	46.4	223 (45.9)	0.125
	Stage 1	60	23.4	72	30.1	132 (27.2)	
	Stage 2	67	27.1	46	19.3	113 (23.3	
	Stage 3	8	3.2	10	4.2	18 (3.7)	
ANY ROP		135	45.6	128	53.5	263 (54.10	0.808
ROP Zone	Zone 1	0		1	0.7	1 (0.4)	
	Zone 2	108	80	114	89.1	222 (84.4)	
	Zone 3	27	20	13	10.2	40 (15.2)	

SpO_2_, oxygen saturation target.

Areas of incomplete peripheral retinal vascularisation were identified in 62 premature infants (12.8%) on images of their last ROP screening visit at term (37–40 weeks PMA). There was a statistically significantly higher prevalence of incomplete peripheral retinal vascularisation at 15.7% (*n* = 39) in Group 1 compared to the Group 2, which had a prevalence of 9.6% (*n* = 23), *p* = 0.043.

Gestational age and birth weight were statistically significantly lower in the premature infants with incomplete peripheral retinal vascularisation compared with those who were fully vascularised [median gestational age of 26 weeks (25–28 weeks) vs. 28 weeks (27–29 weeks), *p* < 0.001, and median birth weight of 872 g (714.25 g–1,002.5 g) vs. 1,150 g (950 g–1,318.75 g) *p* < 0.001], as seen in Table 3.

**Table 3:** Demographic for infants with IPAR.

	Group 1 Low SpO_2_ target	Group 2 High SpO_2_ target	Total	*p*-value
Proportion of full cohort *n* (%)	39/247 (15.7%)	23/239 (9.6%)	62/486 (12.8%)	0.043
Male *n* (%)	18 (46.2%)	9 (39.1%)	27 (43.5%)	
Female *n* (%)	21 (53.8%)	14 (60.9%)	35 (56.5%)	
GA in weeks median (IQR)	26 (25–27)	26 (24–29)	26 (25–28)	0.604
BW in grams median (IQR)	830 (719–1,000)	900 (700–1,010)	872.5 (714.25–1,002.5)	0.406
PMA at worst ROP median (IQR)	33 (32–36)	35 (32–36)	34 (32–36)	0.638

GA, gestational age; BW, birth weight; PMA, post-menopausal age; IQR, interquartile range.

ROP status at the worst stage of ROP among the 62 premature infants with incomplete peripheral retinal vascularisation is detailed in Table 4. The ROP stages upon the last examination showed 90.3% with stage 1 ROP whilst 9.7% had regressed stage 2 ROP. In Group 1, all infants with an incomplete peripheral retinal vascularisation area had persistent stage 1 ROP. In Group 2, 17 (74%) had persistent stage 1 ROP and 6 (26.1%) had persistent regressed stage 2 ROP (Table 5).

**Table 4:** Features of acute phase ROP in infants with IPAR.

	ROP Staging			Vascularity		
	1	2	3	Zone 1	Zone 2	Zone 3
Lower SpO_2_	6 (15.4%)	27 (69.2%)	6 (15.4%)	0	37 (94.9%)	2 (5.1%)
Higher SpO_2_	7 (30.4%)	12 (52.2%)	4 (17.4%)	1 (4.3%)	20 (87%)	2 (8.7%)
Total	13 (21%)	39 (63%)	10 (16%)	1 (1.6%)	57 (92%)	4 (6.4%)

SpO_2_, oxygen saturation target.

**Table 5:** ROP severity and location between the two groups at term.

	ROP Staging		Zone	
	1	2	2	3
Lower SpO_2_	39 (100%)	0	8 (20.5%)	31 (79.5%)
Higher SpO_2_	17 (74%)	6 (26.1%)	7 (30.4%)	16 (69.6%)
*p-*value			0.378	

SpO_2_, oxygen saturation target.

The location of incomplete peripheral retinal vascularisation was zone 3 in 75.8% of affected infants and zone 2 in 24.2%. The extent of the incomplete peripheral retinal vascularisation area had a median of 3 clock hours for both groups (both with a range 1–9 clock hours, *p* = 0.604).

## Discussion

This study reports the prevalence of incomplete peripheral retinal vascularisation in premature infants who had mild ROP that did not require treatment. The prevalence of incomplete peripheral retinal vascularisation at term in this cohort was 12.8%, a significant proportion of infants screened for ROP. Low gestational age and birth weight are known risk factors for the development of ROP (2–4). In our study, the infants with incomplete peripheral retinal vascularisation had statistically significant lower gestational age and birth weight as compared with premature infants without incomplete peripheral retinal vascularisation, indicating that these may be risk factors for the development incomplete peripheral retinal vascularisation.

Higher oxygen saturation levels have been shown to increase the survival of premature infants (16, 17), but are linked to an increased incidence and severity of ROP (7, 8). Our data did not show a difference in ROP location or severity between the lower SpO_2_ group and higher SpO_2_ group. However, there was a statistically significantly higher prevalence of infants with incomplete peripheral retinal vascularisation at term who had been exposed to lower SpO_2_ targets. On the other hand, we found more infants with incomplete peripheral retinal vascularisation in zone 2 in the higher SpO_2_ group, which may indicate a possible association between higher SpO_2_ target and cessation of retinal vascularisation in premature infants which requires further study.

Incomplete peripheral retinal vascularisation has previously been studied in both adults and children. Rutnin and Schepens (18) determined that the vascular termini in adults is 0.5 disc diameters (DD) from the ora serrata. Blair et al. (19), however, observed that in children up to 13 years of age, the vascular retina normally extended to within 1.5 DD or less from the ora serrata. It was concluded that a distance of greater than 2 DD from the ora to the vascularised retinal margin should be considered as abnormal (19). These studies indicate that the retinal vasculature does not always reach the ora serrata in normal eyes; therefore, the residual avascular retina in infants who have a history of ROP, especially those who have avascular retina in zone 3, may be clinically insignificant and considered normal. However, incomplete peripheral retinal vascularisation observed in zone 2 indicates that there is larger area of avascular retina which is certainly not normal. ICROP 3rd edition has defined persistent avascular retina (PAR) and indicated that PAR located in posterior retinae was common in infants following anti-VEGF treatment for severe ROP (20). Long-term follow-up of PAR is recommended due to the risk of retinal hole or retinal detachment (21, 22). Incomplete peripheral retinal vascularisation and PAR is interchangeable in our view as there was no clear time definition to qualify the diagnosis of PAR in ICROP 3rd edition.

The natural history of retinal vascularisation in ROP cases that do not meet the criteria for treatment is unknown, and some of these patients may never fully vascularise. Ho LY et al. reported two similar cases with PAR in zone 3 stage 2 ROP (23). These infants were followed up to 58 weeks post menstrual age and they then received prophylactic laser treatment due to the perceived risk of retinal neovascularisation and attendant complications (23). An avascular retina is prone to retinal thinning, holes, and lattice-like changes and may be associated with retinal detachment later in life (11, 21, 22, 24). Prophylactic laser treatment could potentially reduce the number of reviews required; however, this must be carefully weighed against the potential ocular and systemic complications of laser treatment with further studies are needed in this area (25, 26). However, currently there is not sufficient evidence to support that laser treatment is superior to mere observation in these patients.

Currently, fundus fluorescein angiography (FFA) is the best way to determine if ischaemia is present in the detected avascular retina. Al-Taie et al. (22) recently studied avascular retina in stage 2 ROP that persisted after 45 weeks PMN. They found peripheral vessel leakage in 5.5% of the eyes studied and recommended performing retinal fundus fluorescein angiography in cases of infants with incomplete peripheral retinal vascularisation and applying laser treatment for those with FFA documented vascular along the border between the vascular and avascular retina.

Limitations of this study include its retrospective nature and relatively small sample size. In addition, our study only used coloured Retcam retinal images, not FFA, to determine the presence of incomplete peripheral retinal vascularisation, and this may miss some of the cases of incomplete peripheral retinal vascularisation in those with pigmented retina. On the other hand, there is a small chance of reporting a higher prevalence of IPAR, as in some ROP cases retinal vascularisation may have further developed if those cases were followed longer (our image analysis was based on images collected at last ROP screening between the 37–45 PMA). A further limitation is that only SpO_2_ targets were analysed, and other factors may have potentially contributed to the development of incomplete peripheral retinal vascularisation. Further study of other aspects of neonatal care that may influence the development of retinal vasculature, such as total hours of oxygen use for each infant, is required.

## Conclusion

Incomplete peripheral retinal vascularisation is commonly detected in preterm infants who have been screened for ROP but required no treatment. Lower gestational age and birth weight are possible risk factors for peripheral avascular retina; a higher oxygen saturation target did not seem to affect the prevalence of incomplete peripheral retinal vascularisation, although it may affect the total area of retinal avascularity. Further research into risk factors associated with peripheral avascular retina, as well as long term outcomes and management, is required.

## Data Availability

The raw data supporting the conclusions of this article will be made available by the authors, without undue reservation.
